# Molecular Consortia—Various Structural and Synthetic Concepts for More Effective Therapeutics Synthesis

**DOI:** 10.3390/ijms19041104

**Published:** 2018-04-06

**Authors:** Anna Pawełczyk, Katarzyna Sowa-Kasprzak, Dorota Olender, Lucjusz Zaprutko

**Affiliations:** Department of Organic Chemistry, Pharmaceutical Faculty, Poznan University of Medical Sciences, Grunwaldzka 6, 60-780 Poznań, Poland; kasik@ump.edu.pl (K.S.-K.); dolender@ump.edu.pl (D.O.); zaprutko@ump.edu.pl (L.Z.)

**Keywords:** hybrids, codrugs, prodrugs, conjugates, drug–drug conjugates (DDCs), antibody–drug conjugates (ADCs), polymer–drug conjugates (PDCs), linker, “Lego” chemistry, green synthesis

## Abstract

The design and discovery of novel drug candidates are the initial and most probably the crucial steps in the drug development process. One of the tasks of medicinal chemistry is to produce new molecules that have a desired biological effect. However, even today the search for new pharmaceuticals is a very complicated process that is hard to rationalize. Literature provides many scientific reports on future prospects of design of potentially useful drugs. Many trends have been proposed for the design of new drugs containing different structures (dimers, heterodimers, heteromers, adducts, associates, complexes, biooligomers, dendrimers, dual-, bivalent-, multifunction drugs and codrugs, identical or non-identical twin drugs, mixed or combo drugs, supramolecular particles and various nanoindividuals. Recently much attention has been paid to different strategies of molecular hybridization. In this paper, various molecular combinations were described e.g., drug–drug or drug-non-drug combinations which are expressed in a schematic multi-factor form called a molecular matrix, consisting of four factors: association mode, connection method, and the number of elements and linkers. One of the most popular trends is to create small–small molecule combinations such as different hybrids, codrugs, drug–drug conjugates (DDCs) and small-large molecule combinations such as antibody-drug conjugates (ADCs), polymer-drug conjugates (PDCs) or different prodrugs and macromolecular therapeutics. A review of the structural possibilities of active framework combinations indicates that a wide range of potentially effective novel-type compounds can be formed. What is particularly important is that new therapeutics can be obtained in fast, efficient, and selective methods using current trends in chemical synthesis and the design of drugs such as the “Lego” concept or rational green approach.

## 1. Introduction

The search for more selective and effective therapeutic strategies stems from the realization that it is not always convenient to use a standard therapeutic treatment formula. Many drugs that have been successfully used are characterized by less than ideal pharmacokinetic and biopharmaceutical parameters. In addition to conventional therapy, the development of molecular biology and genetic engineering offers a great contribution, especially in cancer treatment. The latest therapeutic trends include associated, combined, or targeted therapy. A great deal of effort is dedicated to research on selective drug delivery systems and drug activation at the target site of drug action [1,2,3].

The search for new active substances in natural raw materials is not ongoing at present, because many bioactive components of plant extracts have already been isolated and identified. Therefore, current research is very often focused on well-known drugs taking into account their interaction with the target molecule. Moreover, much effort is directed to obtaining new active compounds that could be candidates for new drugs of desired therapeutic index. Development of useful therapeutic chemical species, both for drugs and their possible carriers, is one of the challenges of modern interdisciplinary medical chemistry.

Recently, using the rational drug design approach, single molecules with dual functionality and/or targets have been developed as new drugs. In the literature, there are many scientific reports on the future prospects for the design of potentially useful drugs [1,2,3,4,5,6,7]. Molecular hybridization (conjugation, association, heterodimerization etc.) has been demonstrated to be a viable and effective approach to the development of novel multifunctional compounds, which are called molecular consortia. Molecular consortia are complex chemical and pharmaceutical structural systems which are obtained in a specific manner with the selected active subunits using the appropriate chemical reactions. Selected molecular components may be combined, both naturally and synthetically, in different ways e.g., *x*-fold times, *n*-membered, directly or indirectly, bridged or overlapped, in stable or unstable modes. They can comprise active molecules which are already relatively well known, e.g., linear, pseudo-linear or branched, cyclic, dimeric, oligomeric or polymeric, symmetrical, non-symmetrical or other fan-shaped molecular formation e.g., umbrella one [8]. The molecular consortia term comes from economic terminology and perfectly fits the idea of structural hybridization. In economics, a consortium is an organization bringing together several entities for a limited time and for a specific purpose. The aim of the consortium is joint action leading to implementation of a specific task and not to constitute a stable economic relationship. Elements forming a consortium can be independent in their existing activities, and in the activities of the consortium they implement common policies covered by the agreement. In this paper, the active structures (entities) or other elements combined in a properly designed complex system, acting as living consortia in the human body, are described.

## 2. Structural Concepts

Over the past few years, such general terms as hybrid, conjugate, and codrug compounds have appeared more frequently in the literature and have been successfully adapted to the field of pharmaceutical chemistry. The meaning of the word hybrid goes back to the tradition of Greek *hybris*. Combining of different, sometimes contrasting features has become a domain of the 21st century. The idea behind the hybrid is to make a new entity (of not fully defined status) joining independent elements or compounds which seem to be incompatible. The hybrid strategy in drug design is an alternative to co-formulating of two or more active substances in one tablet and to the classical cocktail therapy based on the administration of a few active substances in separate tablets. This strategy aims to create new pharmacological activity with far more favorable biological effectiveness. This design approach in the search for bioactive molecules is a result of the molecular hybridization of two or more chemically active structures [6,9]. The word conjugated comes from the Latin word *conjugare*, which means mix or match. Chemical conjugation, which is of interest to rational medicinal chemistry, involves using the chemical reactivity of selected biologically active individuals and also drug- or non-drug linkers or carriers. Multifunctional compounds are designed as hybrid or conjugated drugs from two or more pharmacophores/drugs which have specific pharmacological action. Codrug (also known as a drug–drug conjugate or mutual prodrug) consists of two or more drug compounds linked to one another via a labile covalent bond, in contrast to inactive prodrugs which are pharmacologically inactive medications that are metabolized into an active form within the body.

It can be observed that the distinction between conjugate, codrug or hybrid type entities mentioned is very fluid and the molecular consortia concept are used in general to express and describe the new combined structures. All the terms conjugate, codrug, hybrid etc. refer to a new form based on a variety of chemical components and the mechanism of induced activity, although their metabolic pathways are not always the same. Incomplete understanding and lack of clear definition of these terms gives rise to confusion in the increasingly numerous studies concerning the design, synthesis, and biological properties of complex chemical species. Therefore, this paper describes attempts to classify and characterize one of the new dominant directions in the design of potential active structures—molecular consortia—for use mainly in the pharmaceutical field. Many authors have tried to classify the synthesized forms, but there is no clear division and there are many convertible synonyms. Table 1 presents one of the most common classifications of compounds of this type. By increasing the number of pharmacophores subjected to chemical integration of chemical frameworks, trimeric and higher extended combinations are known in the literature as multitarget drugs or multiple ligands [9,10,11]. The most popular and the most frequently reported framework combinations are dual connections, which are completely non-specific structural entities that open up completely new therapeutic perspectives. This paper mainly focuses on combinations of two forms.

This strategy mentioned above leads to various novel combined compounds whose variety of nomenclature in references is different (Table 2).

Twin and triple drugs are defined as compounds that contain two and three pharmacophore components, respectively, exerting pharmacological effects in a one molecule. A twin drug bearing the same pharmacophores is a symmetrical twin drug (homodimer), whereas the one possessing different pharmacophores is a non-symmetrical twin drug (heterodimer) [10,12]. In general, the symmetrical twin drug is expected to produce more potent and/or selective pharmacological effects compared to single molecules, whereas the non-symmetrical twin drug is anticipated to show both pharmacological activities stemming from the individual pharmacophores (dual action) with an additional synergic effect. In a triple drug the two identical portions could bind the same receptor sites simultaneously, while the third portion could bind to a different receptor site or enzyme [12].

**Figure 1:**
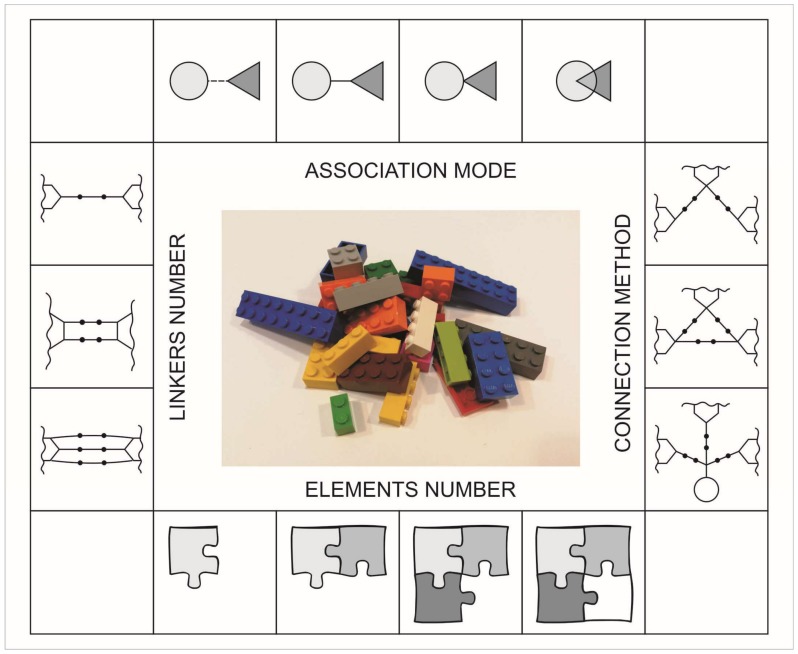
Molecular matrix for “Lego” chemistry approach.

In the dual structures showed in Table 1, the main goal of framework combinations is to design two selected separate active molecules in one skeleton. A possible combination of active compounds can be produced by an indirect or direct method, and depending on degree of frameworks integrated, can be viewed as: linked (linked mode), fused (no-linker mode) and merged (overlap mode) [10,11,12,13]. The general scheme of options for drug-drug or drug-non-drug combinations is given as a multi-factor form called a molecular matrix, consisting of four factors: association mode, connection method, and the number of elements and linkers (Figure 1).

The matrix presented here is a box with blocks, which contains various single elements that can be combined in different ways. The number of possibilities is determined by the availability of various elements and connectors appearing in the box set (molecular matrix). In designing new compounds, we use the factors included in the proposed matrix and consider the following possibilities of connecting them.

- Association mode—describes a type of structural connection, interrelation between different selected elements, kind of attachment (direct or indirect) or structural cross-relationship (linked, fused, merged)
- Connection method—selected chemical and pharmaceutical units, depending on the functionality and reactivity, can form linear, pseudolinear or branched, smaller or larger, new structures
- Elements number—the most popular are structures built from two elements (units, puzzles, segments) but there can be many more of them
- Linkers number—the number of intermediaries between selected elements in the structural population.

Examples of various structural combinations are given in the Figures in the text. They illustrate different combinations of both small-small as well as large-small molecules and different types of connections: direct or indirect (linker mode) and merged ones, in the typical hybrid compounds.

### 2.1. Drug–Drug Conjugates (DDCs)/Codrugs

#### 2.1.1. Linker Concept

Selected components are integrated by a properly designed distinct linker group between two compounds forming a conjugate system. The choice of the right linker and the right method of attachment is the crucial part of conjugate designing [11]. Linkers are classified to different categories according to the mechanism of drug release and drug stability in blood circulation system, including:

- cleavable
- non-cleavable.

Cleavable linkers rely on the physiological environment of the body metabolism (concentration of various agents, pH, presence of enzymes) and disintegrate into two molecules which could then operate independently at separate biological targets. Degradable linkers are often based on the ester linkage, which is easily hydrolysable by an esterase present in the plasma. Chain alkyl or aryl fragments, which may contribute to the additional or increased activity of the whole molecule are used as stable linkers. The linker chemistry determines the release profile of the active ingredients, and thus is crucial to the safety and efficacy of conjugates. Moreover, both types of conjugates (stable and cleavage) are sometimes referred to as intermediate or linked hybrids [11,14]. Linkers are usually small, at least bifunctional reactive molecules, necessary for the chemical integration of different active substances. Any linkers used should allow attachment of the drug without causing inactivation of the bonded parts. Linkers are divided into:

- difunctional
- trifunctional
- crosslinkers.

Their nature can be:

- hydrophilic
- hydrophobic.

Furthermore, the following stand out:

- homofunctional
- heterofunctional (contain diverse functional groups).

The most common functional groups are primary amines, sulfhydryls (thiols), acids, alcohols, or bromides. The linker group can be:

- linear e.g., methylenic or polymeric chain
- non-linear e.g., aromatic, heteroaromatic or non-aromatic cycle
- all the linkers can be available as mono-protected (Boc, Fmoc, and Cbz).

**Figure 2:**
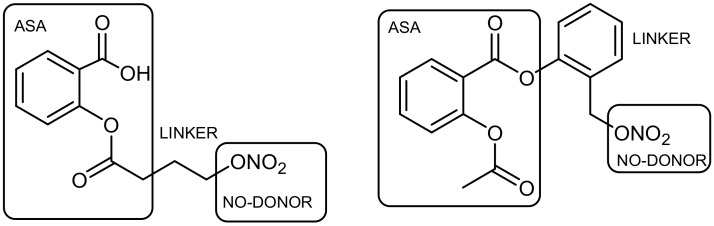
NO-donor aspirin-like linked compounds.

However, in some different cases, the resulting hybrids, which are cleavage conjugates, can act as mutual prodrugs disintegrating on individual components [15,16]. Therefore, it is important to design a suitable—stable or unstable—linker that defines the pharmacological action. Interesting properties, particularly in terms of the safety of use of nonsteroidal anti-inflammatory drugs (NSAIDs) , can be seen in the aspirin molecule (ASA), which is modified by donors of nitric oxide. Aspirin-NO-donors hybrid structures are well tolerated and are more effective in inhibiting tumor cell growth than the standard molecule of aspirin, and even eliminate some side effects [10] (Figure 2).

The other organic hybrid compounds with anti-inflammatory properties have been described extensively in the review by Mohsin and Ahmad [17].

**Figure 3:**
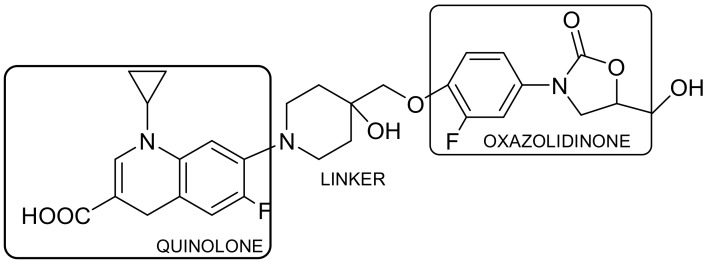
Oxazolidine-quinolone hybrid (Cadazolid).

The oxazolidine-quinolone derivative is promising drug substances for the treatment of infections, exhibiting both antibacterial and against tuberculosis activity [18] (Figure 3).

**Figure 4:**
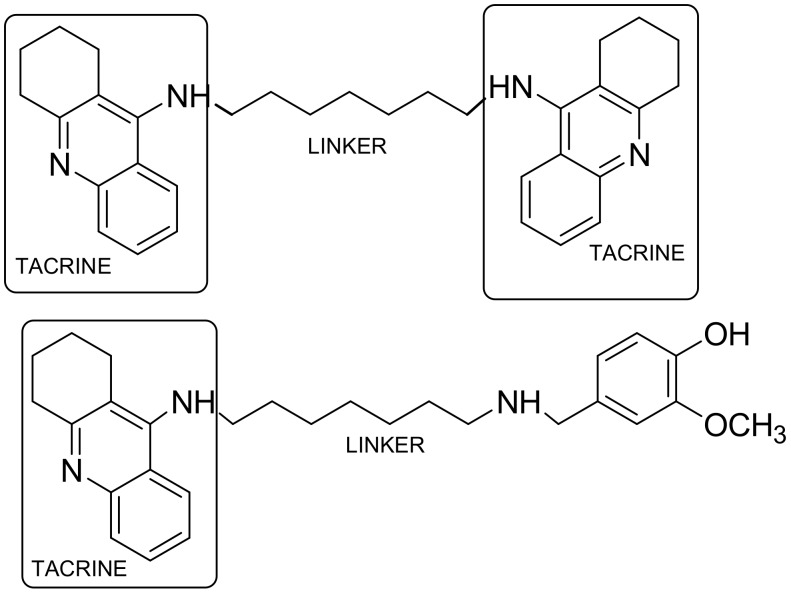
Tacrine homo- and heterodimer.

Tacrine, an acetylcholinesterase inhibitor used for the treatment of Alzheimer’s disease [19], has been dimerized by the polymethylenic chain, leading to more potent and selective bis-tacrine derivatives—the identical twin drug (homodimer) [14,20]. Combining tacrine with a suitably substituted phenyl ring by means of an alkyloamine linker results in heterodimer-type compounds [14] (Figure 4).

#### 2.1.2. No-Linker (Fused) Concept

**Figure 5:**
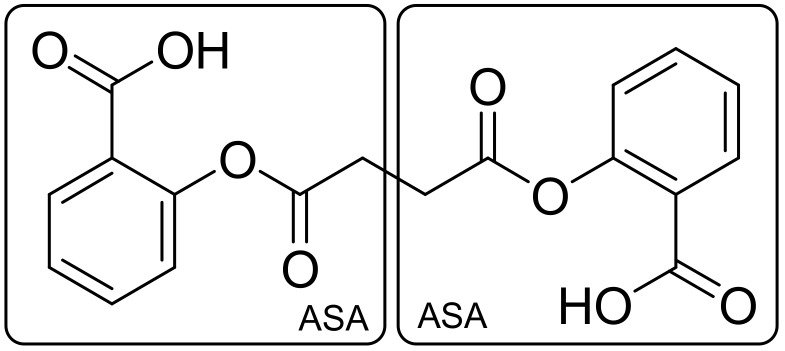
Aspirin—homodimer.

The second approach to connecting frameworks involves direct combination of molecules (fusion) without the use of a linker [10,11]. The pharmacophores of both components do not overlap. Directly fused compounds have the size of the linker decreased such that the frameworks of the pharmacophores are essentially touching, which can be perfectly seen in the case of duplications of aspirin. The result is a combination leading to an identical twin drug called diaspirin, where the connection method is the no-linker mode [10] (Figure 5).

**Figure 6:**
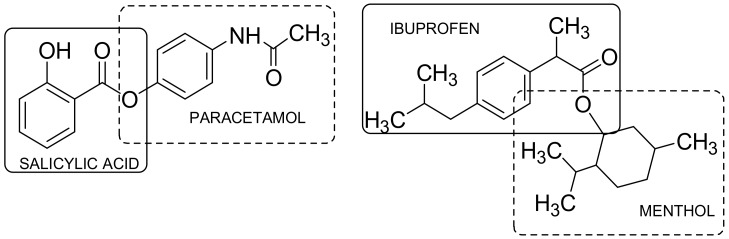
Direct hybrids/codrugs.

Fused particles are usually obtained by combining two different active compounds using the direct reactivity of functional groups of selected pharmacophores. Depending on the chemical nature of the molecules used and the type of connection formed between them, the compounds can be divided into fused stable compounds, which do not decompose in the organism and act as multifunctional innovative drugs through more than one mechanism, and the unstable ones that in physiological conditions decompose to the initial molecules [10,11]. Salicylic acid and paracetamol structures can be integrated to give a non-identical twin drug called acetaminosalol (Figure 6) [9]. Moreover, due to the occurrence of undesirable effects as broad gastrointestinal disorders, ibuprofen has also been chemically modified using a hydroxyl group of eugenol, thymol, and menthol, which leads to an ester association with increased lipophilicity and compounded activity and exerts protective action on the gastric mucosa [21] (Figure 6).

**Figure 7:**
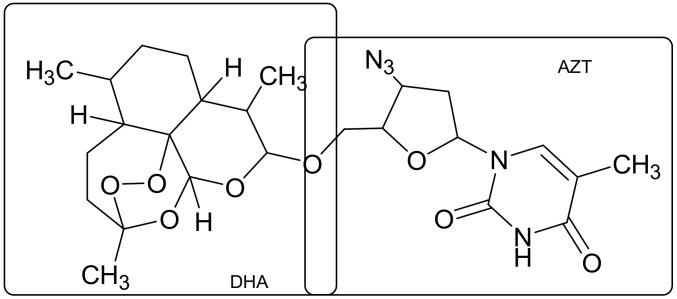
Azidothymidine-dihydroartemizine hybrid/codrug.

A potential drug for the treatment of concomitant diseases such as HIV and malaria is covalent connection of antiretroviral azidothymidine (AZT) and antimalarial dihydroartemizine (DHA). The resulting combination has very good anti-HIV activity, good antimalarial activity, and has a better overall pharmacokinetic profile than DHA [22] (Figure 7).

Based on the above examples, it was observed that fusion of both structures leads to mutual structure integration, but to a very limited extent of one common atom. The overlapping of fragments of structures is characteristic of the next type of hybrid—the so-called merged hybrid.

#### 2.1.3. Overlap (Merged) Concept

The most innovative and consistent structures are merged compounds, containing in their structure selected fragments of the two active substances and some of which have been shared or fused. Frameworks of pharmacophores are merged together by maximizing the degree of overlapping of the skeleton parts to produce smaller and simpler molecules [10,11].

The structural properties of merged curcumin and thalidomide [23] (Figure 10) have led to a series of compounds with interesting properties, of which two have a potent cytostatic activity. Thalidomide, infamously used as an anti-nausea drug for pregnant women, was used once again in the nineties as a drug for the treatment of multiple myeloma. Curcumin, which is a component of the natural extract used as a popular Indian curry spice, has been extensively studied as a potential active substance against cancer, arthritis, and Alzheimer’s disease [24]. However, their potential applications are limited due to the rapid degradation of thalidomide in the organism and its side effects, and due to the limited solubility of curcumin in water. Combination of these two structures has improved the therapeutic efficacy in the treatment of multiple myeloma, and the thalidomide-curcumin hybrid is more effective than each of these substances used separately.

**Figure 8:**
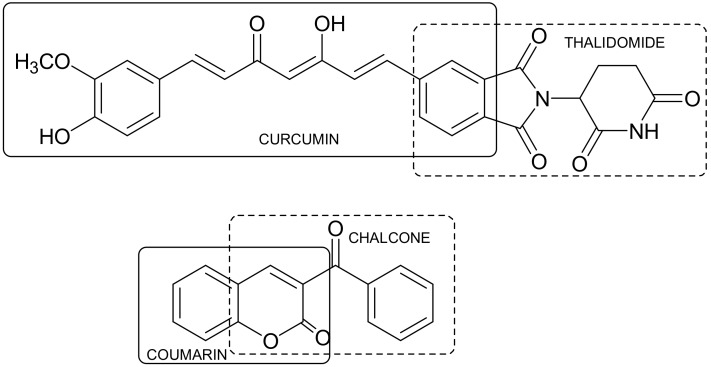
Thalidomide-curcumin and coumarin-chalcone hybrids.

Numerous examples of synthetic modifications and the development of structural and pharmacological integration have been achieved for the antioxidants, which are tested as potential active compounds for the treatment of a number of diseases, such as neurodegenerative disorders, cardio-vascular, inflammatory and neoplastic processes whose genesis is connected to some degree with the action of reactive oxygen or nitrogen, causing the occurrence of oxidative stress [25]. A structurally similar connection is a hybrid containing both structural domain coumarin and chalcone, which is a result of expanding the chalcone structure by means of an additional aromatic ring [26,27] (Figure 8). These types of connections have a higher antioxidant capacity than the well-known antioxidants such as quercetin and catechin and exhibit other promising therapeutic properties [28].

Furthermore, fragment-based drug design (FBDD) strategies have emerged as highly suitable for multitarget drug discovery [29]. Small low-mass fragments are particularly favorable starting point, since they might bind to multiple biological targets due to their chemical simplicity and be grown into lead-like molecules by stepwise addition of functional groups. Against this backdrop, over the years consensus has grown regarding using fragment-based drug discovery (FBDD) as optimal starting points for multitarget drug design. In some ways, the FBDD approach is the antithesis of framework combination as these two cover different regions of the chemical space. In pursuit of novel drug candidates, both framework combination and FBDD strategies, have been adopted. Both methods lead to preliminary interesting results and these approaches are not mutually exclusive but are rather complementary [30].

**Figure 9:**
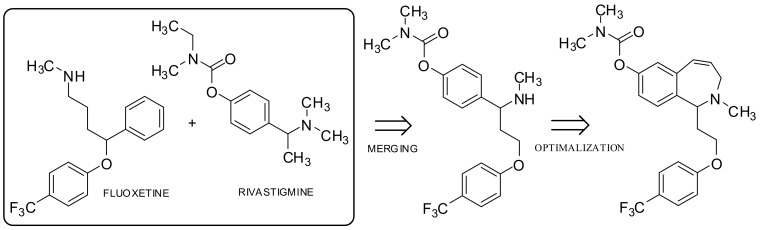
Rivastigmine-fluoxetine hybrid.

Therapeutically active compounds are obtained by merging structures through similar elements belonging to both molecules. This is particularly effective if both substances share several structural components, such as heterocyclic rings or functional groups. This scheme has been used in the design of the dual inhibitor acetylcholinesterase (AChE) and the serotonin protein transporter (SERT). Analyzing the activity of the skeleton and activity of rivastigmine, an AChE inhibitor, it was observed that in order to introduce a hydrophobic component interacting with SERT, rivastigmine should be hybridized with the antidepressant fluoxetine by use of a phenoxyethyl fragment and optimize the structure should be optimized by introducing a rigid seven-membered ring. The resulting hybrid has a dual affinity to both molecular targets (Figure 9) [31].

### 2.2. Antibody-Drug Conjugates (ADCs)

**Figure 10:**
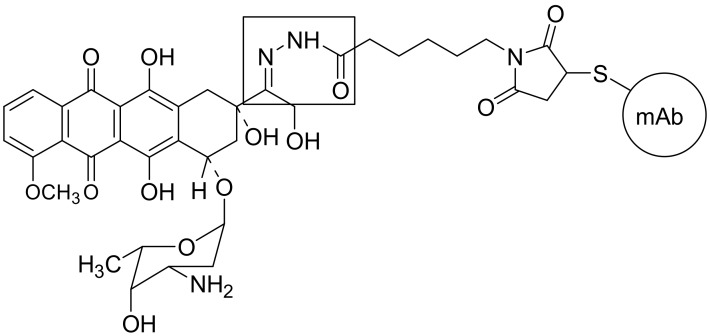
Structure of BRD96-doxorubicin ADC.

Furthermore, thropic molecules such as antibodies, sugars, peptides, or proteins which are responsible for the recognition of receptor sites within the cells or target cells and able to selectively bind to receptors or antigens are also considered for the role of potential drug carriers and active transport [32,33]. Antibody-drug conjugates can be an important class of highly potent biopharmaceutical agents that combine the targeting ability of monoclonal antibodies (mAbs) with small-molecule drugs. Because of their unique nature as biological-small drug hybrids, ADCs are a challenge to develop, from both the scientific and regulatory perspectives. Modification opens the possibility of a new therapeutic strategy for the use of monoclonal antibodies and their tendency to form immunoconjugates with drugs. Antibodies are proteins secreted by the type of plasma cells during an immune response. They are characterized by their ability to specifically recognize antigens and bind with them. Their immunoconjugates comprise a combination of such monoclonal antibodies with anti-cancer drugs [33,34], which due to the increased expression of the receptor and the presence of specific antigens on tumor cells, may be supplied selectively and with high probability to the tumor cells by increasing the intracellular concentration of the drug. A large-molecule antibody is linked to highly potent small molecules (known as: API, drug, payload) that are released only once the antibody binds to the target. The complete ADC must be stable during storage and throughout the time during which the antibody circulates in the organism. The types of conjugation chemistry that can be used are largely determined by the available nucleophilic functional groups such as NH_2_-group of lysine or SH-group of cysteine that are present on the surface of the antibody and are sufficiently distant from the binding site so as not to interfere with the function of the antibody. Maleimide chemistry has been the mainstay for linkage to cysteines. There are two common maleimide-type linkers: maleimidocaproyl and maleimidomethyl cyclohexane-1-carboxylate. Most commonly, cysteines and lysines are reacted with hydrazones, disulfides, peptides and thioether derivatives [35,36]. The perfect linker can guarantee sufficient stability of the cytotoxic conjugate during circulation in the blood stream, effectively preventing premature drug release and facilitating the liberation of the payload at the target tumor cells. Too stable linkers result in low potency and reduced efficacy, while on the other hand, instability of the linker circulation causes poor targeting and high systemic toxicity similar to that of unconjugated therapeutics. Therefore, considerable efforts are being made to design appropriate linkers. Both non-cleavable (antibody degradation after ADCs internalization e.g., thioether linkers) and cleavable linkers play significant roles in determining the pharmacokinetic properties and final therapeutic index. The most typical cleavable linkers used are [35,36]:

Chemically labile linkers:

- acid-cleavable linkers (pH sensitive) e.g., hydrazone (Figure 10) [35]
- reducible linkers (glutathione-sensitive) e.g., disulfide linkers.

Enzyme cleavable linkers:

- peptide-based linkers (protease-sensitive)
- l-glucuronide linkers (glucoronidase-sensitive).

New disulfide linkages and quaternary ammonium salts are recognized as new promising linkers linking the antibody to the drug. They sometimes occur even in one molecule [36].

**Figure 11:**
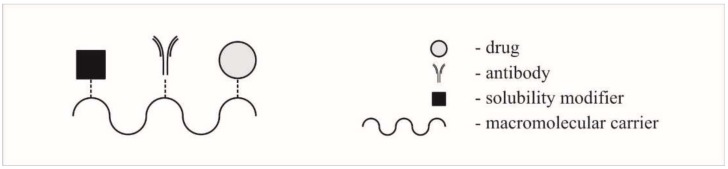
General model of bioconjugate.

A random conjugation process generates a heterogenous mixture of conjugate species and the mixture obtained is generally characterized by an average drug-to-antibody ratio (DAR) [37]. Another interesting solution is complementary antibody-directed enzyme prodrug theraphy (ADEPT) [38,39], in which antibodies are used for transferring enzymes, which then release the drug from an inactive prodrug but only inside the target cell. The general bioconjugate model considers the comprehensive capabilities of conjugation active molecules, antibodies, and contains a solubility modifier, as shown in Figure 11 [16]. Nowadays, antibody-drug conjugates (ADCs) are one of the fastest growing classes of oncological therapeutics [40].

### 2.3. Polymer-Drug Conjugates (PDCs) (Prodrugs)

Currently, one of the most popular trends is to create different macromolecular therapeutics [41,42,43]. This term describes several distinct classes of agents, including polymeric drugs, polymer-drug conjugates, polymer-protein conjugates, polymeric micelles to which the drug is covalently bound [16,38]. Conjugation of the polymer with the active molecule by means of covalent bonds causes a change in the physicochemical and pharmacokinetic properties of the drug and modified polymer features. Particular developments in this area are observed in the studies performed to improve anti-tumor therapy by the targeted delivery of the drug to improve its distribution in the human body and efficient delivery to a specific organ or tissue only, completely excluding normal cells [43]. Small cytostatic molecule, despite their high toxicity, short half-life, low selectivity, and numerous side effects of the molecules, still play invaluable role in cancer therapy. The introduction of this type of intelligent system helps to improve the effectiveness of drugs used and minimizes the side effects induced. Therefore, a significant progress in research on non-toxic and biodegradable polymers metabolizing in the body, which after conjugation with the active molecules lead to an expanded range of potential biocompatible and non-toxic prodrugs, has contributed to the possibility of controlling dosage and active substance release. The best-known polymer proposed for such applications is polyethylene glycol (PEG) and the modification of pharmacologically active agent structures by means of PEG is known as the pegylation process [44,45,46].

This process has already been used in several registered medicinal products among which are conjugate-type products, for example pegylated L-asparaginase [47] used in the treatment of acute lymphoblastic leukemia or pegylated recombinant interferon alpha-2b and alfa-2a applied in the treatment of hepatitis C [45,48]. In addition to polyethylene glycol, vinyl polymers (HPMA) or polyaminoacids and other polymers have been used for drug bioconjugation in both natural and synthetic macromolecules, including peptides, proteins (transferrin), polysaccharides (dextran). The active molecules with reactive chemical groups, such as hydroxyl, amine or carboxylic easily create new prodrugs by forming biohydrolytic connections dependent on the chemical nature of the conjugated units, most commonly of the ester or amide type [16,43,49,50].

The connection of active molecule with the polymer scaffold can be of the following types.

- No-linker mode polymer-drug conjugates obtained by cross-reactivity of functional fragments of pharmacophore present in the components, which leads to the formation of a new covalent bond
- Linker mode polymer-drug conjugates obtained by combining the drug and the polymer carrier by appropriately designed linker.

**Figure 12:**
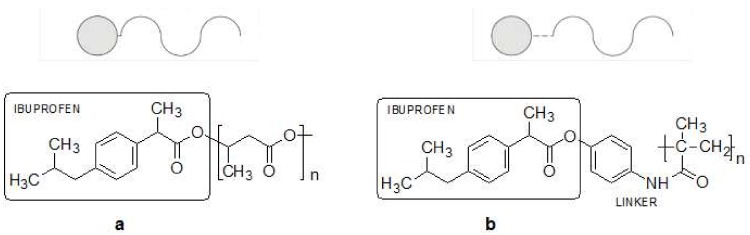
Polymer-drug conjugates (PDCs); (**a**) no-linker mode, (**b**) linker mode.

The resulting conjugates can have a biodegradable or metabolically unstable nature, which allows the release and activation of specific therapeutic substance at the target sites in the body. They act as prodrugs (precursors of drugs), inactive or slightly biologically active substances, which are converted by metabolic processes to active drugs. Selected examples include drug conjugates from the non-steroid group of anti-inflammatory drugs (NSAIDs), such as ibuprofen, aspirin, ketoprofen, naproxen and diclofenac, either in the direct form of ester of ibuprofen conjugates with totally non-toxic oligo (3-hydroxybutyrate) (Figure 12a) [51] or as intermediate amide-ester ibuprofen conjugates with polymethacrylic acid through p-aminophenol linking the drug with the polymer [52] (Figure 12b).

The concept of targeted pharmaceuticals is based on the coordinated interaction of several components: pharmaceutical agent, targeting moiety, pharmaceutical carrier used to load many drug molecules per single targeting moiety, and a target. Different reactive and biocompatible soluble polymers can be used as soluble carriers, whereas the family of insoluble carriers includes microcapsules, nanoparticles, liposomes, micelles. Direct coupling of a drug to a targeting moiety seems the simplest way to prepare a targeted drug. Two basic requirements should be realized in the design of nanocarriers to achieve effective drug delivery. Firstly, drugs should be able to reach the desired tumor sites after administration with minimal loss to their volume and activity in the blood circulation system. Secondly, drugs should only kill tumor cells without harmful effects to healthy tissue. These requirements may be met using two strategies: passive and active targeting of drugs [53]. Passive targeting takes advantage of the unique pathophysiological characteristics of tumor vessels, enabling nanodrugs to accumulate in tumor tissues. Typically, tumor vessels are highly disorganized and dilated with a high number of pores, resulting in compromised lymphatic drainage. One of the earliest nanoscale technologies for passive targeting of drugs was based on the use of liposomes. Moreover, the microenvironment surrounding tumor tissue, is different from that of healthy cells, which is a physiological phenomenon that also supports passive targeting. One way to overcome the limitations of passive targeting is to attach affine ligands (antibodies, peptides or small molecules that only bind to specific receptors on the cell surface) to the surface of the nanocarriers by a variety of conjugation chemistries. Nanocarriers will recognize and bind to target cells through ligand–receptor interactions by the expression of receptors on the cell surface. Based on the receptor-mediated mechanism, targeting conjugates bind to their receptors first, followed by plasma membrane enclosure around the ligand–receptor complex. The newly formed endosome is transferred to specific organelles, and drugs could be released by acidic pH or enzymes.

The use of various types of macromolecular drug carriers, specially designed nanoparticles, oligomers, dendrimers, or polymers has proved to be a major advance, because in many cases the macromolecular bioconjugates obtained exhibit significantly greater efficiency and selectivity than low-molecular therapeutic drugs. In contrast, the same physical incorporation of the active substance in a polymer matrix, which is also of great importance in the design of innovative pharmaceutical forms, does not lead to the conjugate formation. It can be concluded that the function of most oligo- and polymer conjugates is to store and transport the active compounds, so that they are inactive on transportation to the therapeutic goal and protected against degradation, oxidation, and other destructive processes.

Table 3 is summarized general characteristics of mentioned above small-small and large-small combined therapeutics [54,55,56] such as: activity, safety, biotransformation, structural elements number, association mode and design possibility.

## 3. Synthetic Hopes in Combined Drug Chemistry

### 3.1. “Lego” Chemistry Concept

Identifying a hit and subsequently a lead structure for further development is an expensive process and novel sustainable, high-throughput methods are a big challenge for medical chemists. The ideal situation would be one in which molecular synthesis were as simple as building a Lego constructions from single blocs and readily available structure units could be easily linked together to final form made of numerous molecules, in just a few steps. The “Lego” chemistry concept seems to offer many benefits in pharmaceutical field [57]. In chemical references the term “click chemistry” appeared a few years ago. It was first coined by Sharpless in 1998, but with references for drug by Kolb and Sharpless in 2003 [58]. This term describe reactions that are based on readily available starting materials and reagents, need no solvent or soft solvent (such as water), are high yielding, wide in scope, lead to formation of by-products that can be removed without chromatography, are stereospecific, simple to perform and give simple product easy to isolate. This concept was developed in parallel with the interest in capabilities for generating large libraries of compounds for screening in discovery research. Several types of reaction have been identified as thermodynamically-favored that lead specifically to one product, such as nucleophilic ring opening reactions of epoxides and aziridines, non-aldol type carbonyl reactions, formation of hydrazones and heterocycles, additions to carbon-carbon multiple bonds, oxidative formation of epoxides, Michael additions and cycloaddition reactions. The “Lego” chemistry constitutes an interesting approach to the synthesis of drug-like molecules that can accelerate the drug discovery process by utilizing a few practical and reliable reactions. Conjugation, bioconjugation, linker chemistry, nanoparticle surface modification, pharmaceutical-related polymer chemistry etc. can be realized [58,59].

### 3.2. Green Synthesis Concept

The idea of Green chemistry is based on the twelve principles proposed by Anastas and Warner [60] in 1998 (Prevention, Atom Economy, Less Hazardous Chemical Syntheses, Designing Safer Chemicals, Safer Solvents and Auxiliaries, Design for Energy Efficiency, Use of Renewable Feedstocks, Reduce Derivatives, Catalysis, Design for Degradation, Real-time analysis for Pollution Prevention, Inherently Safer Chemistry for Accident Prevention). This idea reduces or eliminates the use or generation of hazardous substances in the design, manufacture, and application of chemical products through the following:

- Application of innovative technology to establish industrial procedures
- Development of environmentally improved routes, synthetic methods, and processes to important products
- Design of new, greener, and safer chemicals and materials
- The use of sustainable resources
- The use of alternatives to chemistry-based solutions
- Development of methodologies and tools for measuring environmental impact
- Development of chemical aspects of renewable energy.

Recently, the environmental impact has become a very important factor in the design of new synthetic methodologies in organic and medicinal chemistry. The use of alternative activation factors, microwaves (MW), or ultrasounds (US) and their mutual cross-combination has become very promising and desirable synthetic methodologies in efficient and fast creation of new drug-like structures [61]. Microwave heating and ultrasonic waves are among the most simple, inexpensive, and valuable tools in applied chemistry. Besides saving energy, these green techniques promote faster and more selective transformations (Table 4).

Examples presented in literature clearly show that combined ultrasound (US) and microwave irradiation (MW), which are practically hazard-free technological innovation that deserves widespread attention in fine-chemical and pharmaceutical research. Although the mechanisms of cavitation and microwave effects are not fully understood, the processes requiring enhanced heat transfer and mass transport will benefit from these green techniques. Combinations of both energies may be simultaneous or sequential and conditions can be tailored for the preparative task. Table 5 shows the parameters characterizing the formation of hydrazides under simultaneous microwave and ultrasounds conditions [62].

### 3.3. Complementary Synergy Concept

“Lego” and green concepts can be additionally supported by a natural additional synergy concept that exceeds the sum of the individual effects of the factors considered [63]. In this paper, synergy effects can be considered on two levels:

- synergism by combining various active structures—pharmacomodulation of two biologically active molecules by chemical hybridization methods leads to a new combined structure with interesting biological activity. According to recent literature [6,7,55] compounds obtained derived from different bioactive molecules often characterized by a synergy of their individual component activities
- synergism by synthesis process intensification by means of combining two non-conventional factors: microwaves and ultrasound. These two effects of process intensification have been used to great effect in various chemical processes and engineering applications. There is certainly scope for them to be applied in the drug chemistry field.

## 4. Conclusions

The first pharmaceuticals were discovered accidentally. Now the task of chemistry is to produce new molecules that exhibit a desired effect, preferably in easy and an environmentally friendly concept. Even today, the search for new pharmaceuticals is a very complicated process that is hard to rationalize. The complexity of developing a new drug requires rational drug design or more precise definition of molecular design. Molecular design can be defined as construction of new molecules with specific chemical or biological activity profiles. The continuous growth of computing power, the advancement of bioinformatics and the development of structural databases of proteins and small molecules make computer-aided drug design a tool of growing importance and increasing use. Modern methods, which do not yet allow a reliable prediction of the final structure of active drugs, make it possible to select promising candidates among the millions of molecules available in the databases and help optimize them for interaction with the receptor. This allows reductions to be made in the cost and duration of the new drug design process. Numerous projects have been undertaken to design and obtain promising compounds for pharmaceutical applications. The identification of a hit and subsequently a lead structure for further development is a very risky and expensive process. In the latest literature, there are many examples of reports on molecular combinations [5,6,24,25,28,64,65,66] of both natural and synthetic pharmacophores, and drug and non-drug scaffolds in order to find the optimal and safest therapeutics with variety bioactivities, in particular: anti-cancer, anti-inflammatory, anti-malarial, anti-tuberculosis agents, potential drugs for Alzheimer’s disease etc. In addition to developing new therapeutics, an important branch of research in the pharmaceutical sector is that concerned with increasing effectiveness of existing molecules. The use of hybrid type molecules, compared with drug combination therapy, brings several benefits, such as increased bioactivity of compounds, reduced risk of drug resistance, improved pharmacokinetic properties with respect to constituent units, and synergistic effects on individual activities that significantly affect their increase or offer completely new and desirable pharmacological potential. A chemical hybrid of two (or more) different and distinctly acting molecules composed of different structural elements responsible for specific pharmacological actions and acting simultaneously or sequentially, may provide a combination of these features, which may improve the drug effectiveness. These structures may be obtained from a variety of classes of organic compounds and usually combine small molecules, although large molecules (antibody, polypeptides, nucleic acids) are also used. A review of the literature reveals the structural diversity of multifunctional structures and indicates a wide spectrum of potential applications of framework connections, especially in multi-factorial diseases therapy by simultaneous, poly-active, and multi-tasking performance along the identified disease pathway. Through a rational combination of already known drugs and synthetic methods and synthetic trends, their mutual synergy of action could lead to a unique strengthening of the pharmacological activity of multifunctional drug molecules.

## Figures and Tables

**Table 1:** The most common types of hybridized compounds.

ASSOCIATION MODE			
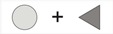			
intermediate		direct	
linker mode		no-linker mode	overlap mode
cleavage linked	stable linked	fused	merged
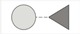	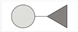	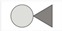	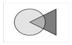
conjugate cleavage	conjugate	fused hybrid/codrugs	merged hybrid (chimera)

**Table 2:** The most common terms for hybridized compounds.

ASSOCIATION FORMS	
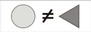	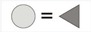
Association	Duplication/Dimerization
**drugs:**	
non-identical twin drugs	identical twin drugs
two-pharmacophore drugs	one-pharmacophore twin drugs
non-symetrical twin drugs	symmetrical drugs
dual acting drugs	
hybrid drugs	
codrugs	
mutual prodrugs	
prodrugs	
**conjugates:**	
drug-drug conjugates (DDCs)	
antybody-drug conjugates (ADCs)	
polymer-drug conjugates (PDCs)	
**other:**	
heterodimers	homodimers

**Table 3:** Comparative characteristics the main type of hybridized compounds.

Molecular Consortia Descriptors	Hybrid Drugs	Conjugates	Codrugs	Prodrugs
Elements number	two (or more) distinct pharmacophore	two (or more) elements (DDCs, ADCs, PDCs)	two (or more) therapeutic compounds	two (or more) elements, only one is bioactive drug, carrier: inactivepolymer, antibody, gene, virus, enzyme
Association mode	direct ^a^	direct ^a^	direct ^a^	direct ^a^
	indirect ^b^	indirect ^b^	indirect ^b^	indirect ^b^
	merged ^c^			
Transformation (In vivo)	no-enzymatic cleavage	linker dependent selected	enzymatic cleavage	enzymatic cleavage
Activity	dual effects, different targets	dual effects, different targets	dual effects from both drugs	single effect (carrier is inactive)
Safety	enhancing efficacy, improving safety	improved therapeutic index	improved therapeutic index	additional toxicity depends on carrier
Design options	based on non-labile linker	based on labile or non-labile linker	based on specific chemical function	unlimited approach

^a^ direct—no-linker concept, ^b^ indirect—linker concept, ^c^ merged—overlap concept.

**Table 4:** Ultrasounds versus microwave.

Reaction Characteristics	Ultrasounds (US)	Microwaves (MW)
Reaction media	aqueous and organic solvents	MW-absorbing liquids; solvent-free protocols
Acceleration	variable (from min to h)	high (min, even seconds!)
Activation	cavitation (thermal effects)	thermal effects, (specific non-thermal)
Scaling up	possible but still a challenge	Possible
Chemical effects	selectivity changes mechanistic switching waste reductions	selectivity changes waste reductions
Other effects	light emission, cleaning, microstreaming	heating above boiling points, change in solvent properties

**Table 5:** Hydrazinolysis of methyl salicylate.

Method	Time	Yield (%)
reflux	9 h	73
US (50 W)	1.5 h	79
MW (200 W)	18 min	80
MW + US	40 s	84
